# Performance of Large Area n-TOPCon Solar Cells with Selective Poly-Si Based Passivating Contacts Prepared by PECVD Method

**DOI:** 10.3390/ma17040849

**Published:** 2024-02-09

**Authors:** Zhaobin Liu, Chunlin Guo, Ya Liu, Jianhua Wang, Xuping Su, Qinqin Wang

**Affiliations:** 1 School of Materials Science and Engineering, Changzhou University, Changzhou 213164, China; 2 Jiangsu Collaborative Innovation Center of Photovoltaic Science and Engineering, Changzhou University, Changzhou 213164, China; 3 Institute of Microelectronics of the Chinese Academy of Sciences, Beijing 100029, China; 4 Institute of Technology for Carbon Neutralition, Yangzhou University, Yangzhou 225009, China

**Keywords:** n-TOPCon, mask, laser, P-SE

## Abstract

Selective emitter (SE) technology significantly influences the passivation and contact properties of n-TOPCon solar cells. In this study, three mask layers (SiO*_x_*, SiN*_x_*, and SiO*_x_*N*_y_*) were employed to fabricate n-TOPCon solar cells with phosphorus (P)-SE structures on the rear side using a three-step method. Additionally, phosphosilicon glass (PSG) was used to prepare n-TOPCon solar cells with P-SE structure on the rear side using four-step method, and the comparative analysis of electrical properties were studied. The SiO*_x_* mask with a laser power of 20 W (O2 group) achieved the highest solar cell efficiency (*E_ff_*, 24.85%), The open-circuit voltage (*V_oc_*) is 2.4 mV higher than that of the H1 group, and the fill factor (*FF*) is 1.88% higher than that of the L1 group. Furthermore, the final *E_ff_* of solar cell is 0.17% higher than that of the L1 group and 0.20% higher than that of the H1 group. In contrast, using the four-step method and a laser power of 20 W (P2 group), a maximum *E_ff_* of 24.82% was achieved. Moreover, it exhibited an *V_oc_*, which is elevated by 3.2 mV compared to the H1 group, and *FF* increased by 1.49% compared to the L1 group. Furthermore, the overall *E_ff_* of the P2 group outperforms both the L1 and H1 groups by approximately 0.14% and 0.17%, respectively. In the four-step groups, the *E_ff_* of each laser condition group was improved compared with the L1 group and H1 group, The stability observed within the four-step method surpassed that of the three-step groups. However, in terms of full-scale electrical properties, the three-step method can achieve comparable results as those obtained from the four-step method. This research holds significant guiding implications for upgrading the n-TOPCon solar cell rear-side technology during mass production.

## 1. Introduction

The TOPCon (tunnel oxide passivated contact) solar cell with a surface area of 4 cm^2^ was proposed and developed by the Fraunhofer Institute for Solar Energy Systems in Germany in 2013, achieving an impressive efficiency of 23.7% [1]. The efficiency (*E_ff_*) was enhanced to 25.7% in 2017 by optimizing the village resistivity and process flow at the Fraunhofer Institute for Solar Energy Systems in Germany [2]. The efficiency of the POLO-IBC solar cell with the TOPCon structure combined with IBC reached 26.1% [3] in 2018. Due to their excellent passivation performance, TOPCon solar cells have been extensively studied in recent years [4,5,6,7,8,9,10,11,12,13,14]. There are many factors that affect the efficiency of solar cells, including surface recombination rate [15] and contact resistance [16]. The surface recombination rate primarily relies on the passivation quality of the surface, while the contact resistance is influenced by the doping concentration in the contact region. As the efficiency of TOPCon solar cells continues to improve, the reduction in frontal contact area has emerged as a predominant factor constraining solar cell performance [17]. A commonly employed technique involves the fabrication of a selective emitter (SE) on the front side [18]. This approach serves two purposes: firstly, it mitigates carrier recombination in the emitter region and enhances surface passivation; secondly, it reduces contact resistivity (*ρ_c_*) in the contact region, thereby augmenting the *FF* [19]. Currently, laser treatment has been shown to increase *V_oc_* by 5–8 mV [20]. The maturity and widespread adoption of positive B-SE technology in industrial production, coupled with the advancement of laser-enhanced contact optimization (LECO) technology, offer potential for further enhancement of *V_oc_* and *FF*, thereby augmenting the *E_ff_* of solar cells. Additionally, the rear-side surface is passivated using SiO*_x_*/Poly-Si(n^+^) [21]. Despite the improved passivation and contact effects, due to the high P atoms doping concentration leading to high recombination, it is still possible to achieve SE through laser technology reducing the P atoms doping concentration in the non-contact region while increasing it in the contact region. Consequently, optimal rear surface passivation and superior rear contact performance are attained.

The principle of laser doping involves focusing a laser beam on the surface of crystalline silicon, resulting in the generation of high temperatures exceeding 1414 °C, followed by a rapid quenching process (~735 °C/s) [22]. Sufficient energy can induce rapid melting and recrystallization in the local area of the wafer surface, with minimal heat generated outside of the laser region. The solubility of doped elements in liquid Si is an order of magnitude higher than that in the solid state. By adjusting laser power, wavelength, and pulse parameters, diffusion or activation of dopants can be achieved [23]. Due to the significant recombination introduced by the frontal contact of n-TOPCon solar cells [17], B-SE technology has garnered increased attention and been extensively investigated by scholars [11]. Employing laser irradiation on the metallized pattern region enables the attainment of selective emitter characteristics, with low doping concentration in the undoped region and high doping concentration in the doped region. Nevertheless, it is worth noting that laser-induced defects, contamination, and discontinuities in selective emitters may also lead to a degradation in solar cells’ performance [24,25]. As is widely recognized, the conventional SE technology involves the laser processing of boron-rich boron-silicate glass (BSG) or phosphate-rich phosphorus-silicon glass (PSG) as precursors, followed by high-temperature annealing and oxidation to form a protective film on the surface. Traditional B diffusion typically requires over 80 min at temperatures ranging from 900 to 1000 °C [26]. Similarly, P annealing oxidation also necessitates approximately 50 min at temperatures between 800 and 900 °C [27]. Consequently, the conventional SE method exhibits prolonged process duration and significant energy loss.

Currently, the P-SE of n-TOPCon solar cells prepared using the PECVD (plasma-enhanced chemical vapor deposition) method can be accomplished through four steps: firstly, preparing SiO*_x_*/a-Si(n^+^) and SiO*_x_*; secondly, depositing the P source to form PSG; thirdly, performing laser treatment (SE); and finally, conducting annealing and oxidation. Alternatively, it can be achieved in three steps: first, by the PECVD preparation of SiO*_x_*/a-Si(n^+^) and a mask layer; then, by laser treatment (opening film and SE); and lastly by annealing oxidation followed by deposition of P diffusion and oxidation. The two laser methods have different effects as the three-step laser involves opening film and SE effects, while the four-step laser only includes the SE effect. The LPCVD (low pressure chemical vapor deposition) method can only be completed using the four-step method due to its inability regarding in situ doping. Investigating various approaches to realize P-SE is highly significant for enhancing the *E_ff_* of solar cells and reducing energy consumption.

## 2. Experiment and Characterization

### 2.1. Experiment

**Figure 1:**
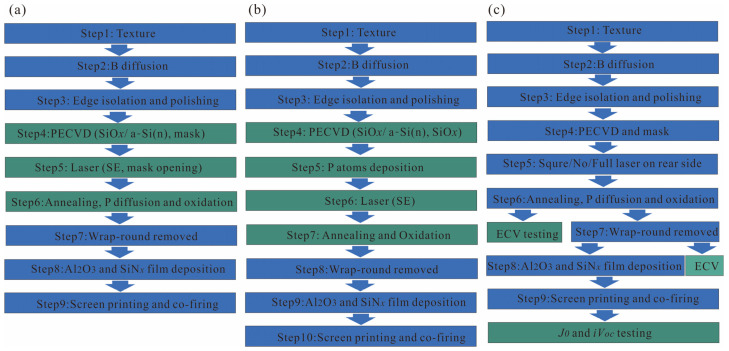
Experimental flow chart: (**a**) three-step SE process; (**b**) four-step SE process; (**c**) sample test flow chart.

In this experiment, 182 × 182 × 0.14 mm^3^ n-type Cz silicon wafers (LONGi Green Energy Technology Co., Ltd., Xi’an, China) with a resistivity of 1.0–1.2 Ω.cm were used to fabricate n-TOPCon solar cells. The preparation process and variables for the n-TOPCon solar cell test samples are illustrated in Figure 1. The three-step method to achieve the SE is shown in Figure 1a. Alkali texturing was used to create a pyramid-like surface structure, enhancing light trapping. The height of the pyramids was controlled to be less than 2 μm, and the reflectivity was maintained at 9.5 ± 0.2%. B diffusion is used to form the PN junction with a sheet resistance of 140 Ω/sq and junction depth of 0.8 μm. The rear side and surrounding BSG layers were removed through HF chain cleaning, followed by polishing with a mixed aqueous solution containing additives and alkali to eliminate the B wrap-round on the rear and edges simultaneously, and in this step, the rear-side reflectance was controlled to be 42.0 ± 0.2%. Subsequently, the tunneling oxidation passivation structure of a-Si(n)/SiO*_x_* was prepared using PECVD (CETC, Changsha, China). An electric field was applied in the reactor, relying on radio frequency induction to ionize the target material source gas and generate plasma, increasing reactant activity for the low temperature deposition (≤450 ℃) process. After evacuating the wafers into the PECVD equipment cavity, leakage detection was performed at 420 ℃. The entire preparation process was carried out at 420 ℃. Firstly, the tunneling oxide layer, SiO*_x_*, was prepared with a flow rate of N_2_O set at 8000 sccm, with the pressure maintained at 300 Pa, power set to 10,000 W, and pulse on/off ratio adjusted to 2 ms/200 ms. Then, an a-Si(n) layer was fabricated for a total duration of 1300 s. The PH_4_ flow rate was adjusted to 1200 sccm, while PH_3_ and H_2_ were introduced at rates of 1000 sccm and 3500 sccm, respectively. The process operated under a pressure of 300 Pa with a power input of 14,000 W, employing a pulse on/off ratio of 4 ms/50 ms. Finally, the mask layer deposition took place over a period lasting for 100 s. During this step, the flow rate of SiH_4_ was 1100 sccm, the flow rate of N_2_O was 6000 sccm, the pressure was 180 Pa, the power was 9000 W, and the pulse on/off was 5 ms/100 ms. After completion of the steps, the tube underwent two cycles of pumping out and nitrogen filling to restore normal pressure, and then the tube was released.

A green nanosecond laser was employed for film opening as well as selective emitter formation, and the spot size of the laser is 120 × 120 μm^2^. The wafers were annealed, P atoms diffused and oxidized; The PSG on the edge was removed through front chain HF cleaning, while a portion of the BSG was retained on the front. The wrap-round was removed through alkali polishing. Alumina passivation was applied to the front, along with silicon nitride passivation on both sides. Metal electrodes were printed and fired to form the final solar cells in this study. The deposition times of SiO*_x_*, SiN*_x_*, and SiO*_x_*N*_y_* were set at 200 s to prevent P diffusion in the lightly doped region after laser treatment. The power, source amount, and deposition temperature for all three types of masks deposited by PECVD were kept consistent. The laser SE powers used were 15 W, 20 W, and 25 W.

Figure 1b illustrates the four-step P-SE process of the n-TOPCon solar cell preparation process, wherein oxidation was required post laser to generate a SiO*_x_* protective layer safeguarding the SE region when the wrap-round was removed. Despite the realization of a selective emitter in the four-step method, only one step corresponds to an effective selective emitter. In the three-step method, although no P atoms exist in the surface mask layer, laser played a role by driving P atoms from the shallow surface layer deeper. Following laser treatment, the surface mask layer was etched away and subsequently, annealing, P deposition, and drive in were conducted. However, due to the blocking effect of the non-laser areas’ mask layers, it was challenging for P atoms to infiltrate poly. Conversely, on the laser-treated region’s surface, P atoms were easily driven into poly as part of forming a selective emitter, thus, constituting the second step involved in this process. Although there are merely three steps involved here, two of them contribute towards establishing a selective emitter.

The test wafers prepared during the experiment are shown in Figure 1c. The laser area of Step 5 was square, full-face, and laser free. The laser block film after Step 6 was utilized for sheet resistance (*R_sheet_*) and the ECV test, while the ECV test was conducted on the wafer after the wrap-round was removed to confirm the cleaning window and compare it with the wafer without the wrap-round removed. *J*_0_ and *iV_oc_* were tested using both full laser and non-laser wafers. The difference between the *J*_0_ of the non-laser wafer and *J*_0_ of the full-face laser wafer at the rear side represents the compound *J*_0_ caused by laser damage. By considering the width of the gate laser slot and number of gate lines, we can calculate the increase in *J*_0_ brought about by rear-side lasers.

**Figure 2:**
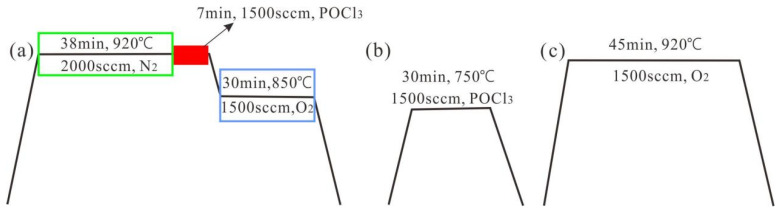
(**a**) Step 6 of the three-step method; (**b**) Step 5 of the four-step laser method; (**c**) Step 7 of the four-step method.

**Figure 3:**
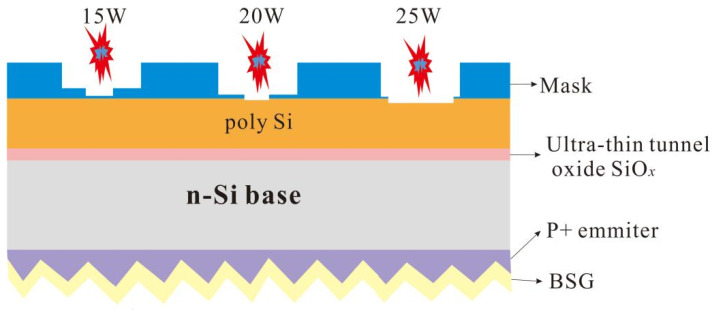
Laser diagram of P-SE process.

Figure 2 shows the schematic diagram of the high temperature diffusion process of the two laser routes. Step 6 of the three-step method raised the temperature to 920 °C first for nitrogen protection and crystallization for 45 min, after which POCl_3_ was fed for the next 7 min, then the temperature was lowered to 850 °C, and then the temperature was lowered after 30 min of oxygen constant temperature. Figure 2b shows Step 5 of the four-step method, where the P atoms were deposited for 30 min at a low temperature of 750 °C; Figure 2c shows Step 7 of the four-step method, where the temperature was raised to 920 °C, and oxygen was injected under constant temperature for 45 min. Figure 3 illustrates a schematic diagram of laser film opening. Table 1 presents all the experiments along with their respective experimental conditions.

### 2.2. Characterization

The rear-side morphologies were examined using an Olympus optical microscope (DSX-HRSU, Olympus Optical Co., Ltd., Tokyo, Japan) under different experimental conditions. Additionally, the *R_sheet_* on the sample’s rear side was measured using a Napson test instrument (RG-2000PV, Napson, Wuxi, Chaina), and the P atoms doping curve was determined under various experimental settings using a WEP Wafer Profiler (CVP21, Saratoga Technology International, Saratoga, CA, USA). The solar cell’s open circuit voltage (*V_oc_*), short circuit current density (*J_sc_*), fill factor (*FF*), series resistance (*R_s_*), and efficiency (*E_ff_*) were evaluated using an IV test instrument (Delta Electronics, Inc., Shanghai, China) under standard testing conditions of 1000 w/m^2^ illumination, temperature of 25 ± 1 °C, 70% ambient humidity, and simulated am 1.5 spectral distribution.

In order to assess the minority carrier lifetime (*τ_eff_*) and implied open circuit voltage (*iV_oc_*), we conducted relevant experiments using the WCT-120 [28] instrument (Sinton Instruments, Sinton, TX, USA). Additionally, the specific contact resistivity of the grid line was measured using the transmission line model (TLM) and GP-4 [29] probe (GP Solar GmbH, Memmingen, Germany) transmission line model method. The *EQE* test was performed utilizing the PVE300-IV test equipment (Enli Technology Co., Ltd., Shenzhen, China), while the current loss analysis relied on the V1.4 calculation tool provided by the Singapore Solar Energy Research Institute [30].

## 3. Results and Discussions

### 3.1. Morphologies Analysis

**Figure 4:**
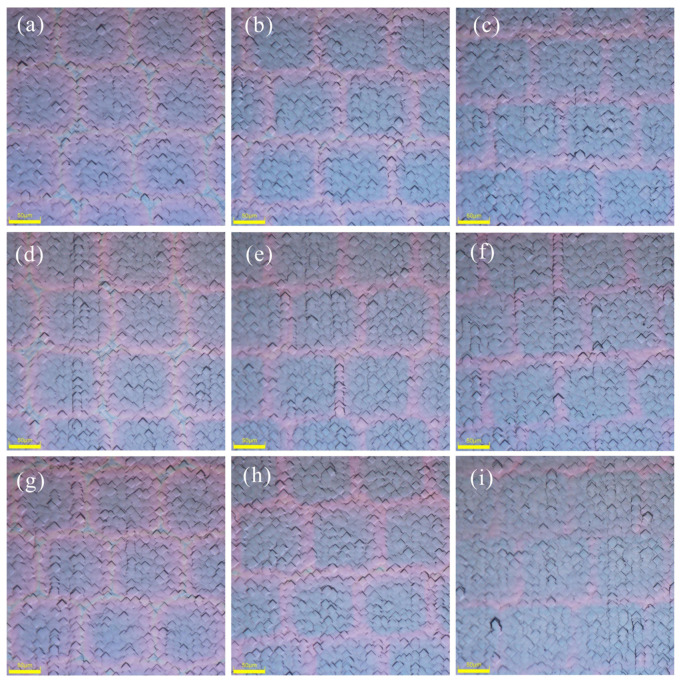
Microscope image of the laser spot on n-TOPCon cell: (**a**) O1; (**b**) O2; (**c**) O3; (**d**) N1; (**e**) N2; (**f**) N3; (**g**) S1; (**h**) S2; (**i**) S3.

After laser treatment under different experimental conditions, the spot morphologies are depicted in Figure 4. By manipulating the laser processing speed and pulse frequency, the laser overlap ratio can be adjusted [31]. In the mass production process, we regulated the slight overlap of spots due to differential cooling rates between the spot’s edge and its middle. Under high power conditions, micro-textures may manifest in the overlapping region [32], leading to a reduction in the minority carrier lifetime of the solar cell. We know that in the mass production process of n-TOPCon prepared using the low-pressure chemical vapor deposition (LPCVD) method, some manufacturers judge the thickness of poly silicon by the color of the front of the wafer after P atoms diffusion and annealing, because the mask layer deposited on different poly silicon thicknesses show different colors. Following the deposition of diverse mask layers through the PECVD method, a light gray hue akin to the wafers is observed on their surfaces. The localized high temperature generated by laser formation induces crystallization of a-Si(n^+^) into Poly-Si(n^+^), which shows a pink coloration on the masked layers, while cyan coloration emerges at spot junctions, particularly noticeable at 15 W power. The 15 W laser produces the darkest color in each mask group, while the 25 W laser produces the lightest color. Additionally, the SiO_x_N_y_ mask group’s 25 W laser creates the lightest color. Due to the presence of the mask layer, Poly-Si with different thicknesses show different colors. The colors disappear after the mask layer is removed, and there are some areas at the edge of the spot where the film is completely opened. Higher power lasers can create cracks or even grooves in open areas [33]. This creates more damage, which reduces the lifetime of a carrier. Previous studies have demonstrated that noticeable defect formations occur only when laser irradiation surpasses the silicon melting threshold, with the recrystallization rate being a crucial parameter for defect generation. Specifically, the surface electrical and optical properties of solar cells are influenced by dislocation density and oxygen incorporation [34,35]. Generally, the types of defects commonly observed depend largely on two factors: (1) initial concentration of external impurities (carbon, oxygen, nitrogen, dopants); and (2) structural defects in the silicon crystal, which directly impact its melting threshold [36].

**Figure 5:**
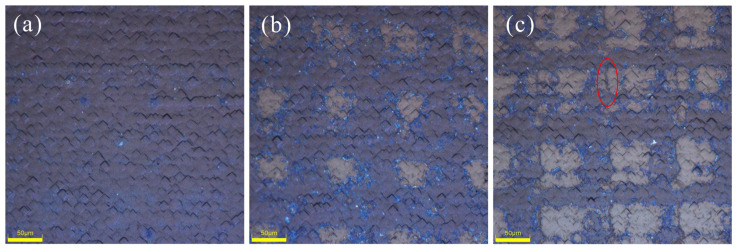
Microscope image after four-step SE laser: (**a**) P1, (**b**) P2, (**c**) P3.

In the three-step process, the microscopic morphology damage of the spot after laser treatment with different masks is not readily discernible, whereas in the four-step process, a more distinct visualization of the state of the surface film layer opening can be observed. As depicted in Figure 5, at a power of 15 W, the laser spot is barely discernible on the surface. However, upon increasing the laser power to 20 W, it becomes evident that the surface film layer Is removed, thereby exposing the wafer directly. Notably, the initial removal by the laser occurs precisely at the center of the spot. Subsequently, raising the laser power to 25 W leads to a further expansion of the PSG removal area. The PSG serves as an effective reservoir for doping diffusion, resulting in highly doped emitters with significant junction depths [37]. When moved from the interstitial position to the substituted position, the inactive phosphorus atoms in the emitter become electrically active [38]. If excessively high laser power is selected, the ablation of the surface is initiated, leading to severe silicon damage that can be visualized under microscopy [39]. As depicted in red circle position of Figure 5c, excessive heat provided by lasers causes the ablation of mask layers and melting on transmitter surfaces, resulting in debris upon solidification on wafer surfaces [40]. Furthermore, extensive dislocations along with micro-twins and swirling defects have been identified [41,42,43,44]. Additionally, SEM imaging has revealed other defects within melting zones when continuous wave (CW) lasers are employed [45]. Other defects in the melting region have been also observed using SEM imaging, and the trap state defects are mainly associated with oxygen vacancy and vacancy [44,45,46,47].

**Figure 6:**
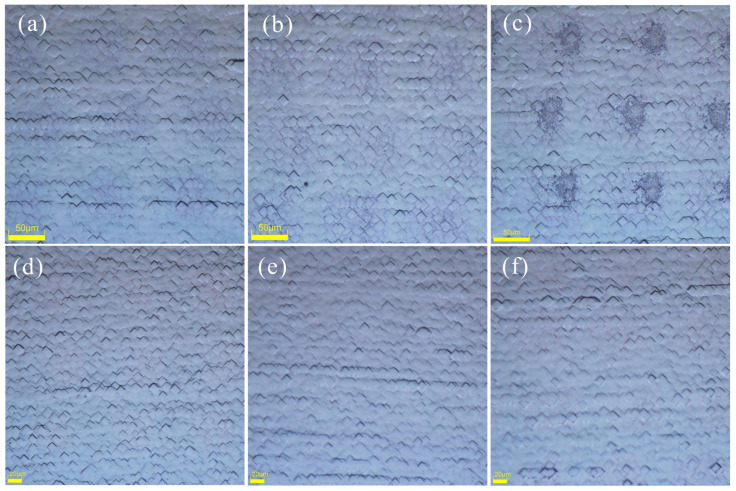
Morphologies of laser region after wrap-round removal and cleaning: (**a**) O1; (**b**) O2; (**c**) O3; (**d**) P1; (**e**) P2; (**f**) P3.

The microscope images after the wrap-round removal and cleaning are presented in Figure 6. We show the SiO*_x_* mask groups in the three-step method, and the images of the four steps after the wrap-round removal and cleaning. As the laser energy increases, the extent of the damage becomes more pronounced, with Figure 6a,b showing slight damage in the center area of the laser spot, while Figure 6c provides a clearer depiction. The laser area of the four-step method may be due to the thick PSG, and the laser spot is indistinct after cleaning. To achieve a higher laser doping concentration through the P-SE process, reliance on PSG as a P source is necessary but limited; thus, increasing the P atoms content within PSG becomes imperative for augmenting the SE’s P source. Augmenting the P content also leads to an increase in the PSG thickness, which subsequently lowers the damage layer during the application of the laser SE technique. Nevertheless, Figure 6f clearly demonstrates the recrystallization occurring on the silicon wafer post melting due to the surface tension effects between liquid phases [25], resulting in a blurred division line at the pyramid base.

**Figure 7:**
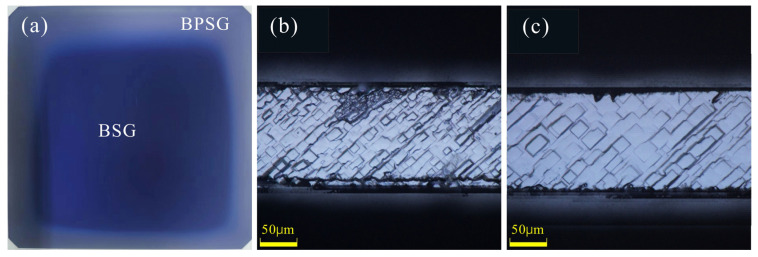
(**a**) BPSG wrap-round; (**b**) Unqualified side; (**c**) Qualified side.

The four-step process of P diffusion annealing after the laser is mainly used for three reasons: firstly, it facilitates the conversion of a-Si(n^+^) into Poly-Si(n^+^); secondly, it enables the deposition and promotion of P atoms to enhance the P concentration in the contact region; thirdly, it facilitates the formation of a protective PSG film. During the deposition process of P atoms and oxidation, BPSG tends to wrap round the front surface of the wafer, as illustrated in Figure 7a. Chain single-sided HF cleaning is required before the wrap-round removal and cleaning, and the corrosion rate of BPSG is much higher than that of PSG. However, a critical aspect of this step is that the corrosion time of BPSG should be longer than that of PSG. If the deposition of P atoms and oxidation takes too long, an increased amount of BPSG is formed, leading to a comparable or shorter corrosion time for BPSG compared to PSG. Consequently, there is no available window for the wrap-round removal and cleaning. In such cases, if the front part of the BPSG is retained during the wrap-round removal and cleaning, it becomes impossible to clean the Poly-Si(n^+^) around the wafer (as shown in Figure 7b), resulting in a pyramid base size on the side remaining unchanged after the wrap-round removal and cleaning. Our observation indicates that after the wrap-round removal and cleaning, an increase in the pyramid base size from its original range of 8–9 μm to more than 13 μm (as depicted in Figure 7c) is required for the complete removal/cleaning of Poly-Si(n^+^)/SiO*_x_*. It should be noted that a certain thickness of a PSG protective layer needs to be prepared in order to ensure Poly-Si corrosion by alkali solution during the wrap-round removal and cleaning. The oxidation time cannot be reduced here, and only the time for deposition of P atoms can be reduced. But if the amount of deposition of P atoms is insufficient, it is impossible to pull apart the concentration difference between the heavily doped region and the lightly doped region, and then a good selective emitter cannot be formed, so the time for P deposition needs to be strictly controlled. We verified that the best P deposition time is 7 min. However, when the deposition time exceeds 10 min, no window is observed for the wrap-round removal and cleaning. Additionally, if the deposition time of P atoms is less than 4 min, the concentration difference between the heavily doped and lightly doped regions falls below 1 × 10^20^ atoms/cm^3^, rendering it incapable of forming a satisfactory selective emitter.

### 3.2. ECV Profiles of Experimental Groups

**Figure 8:**
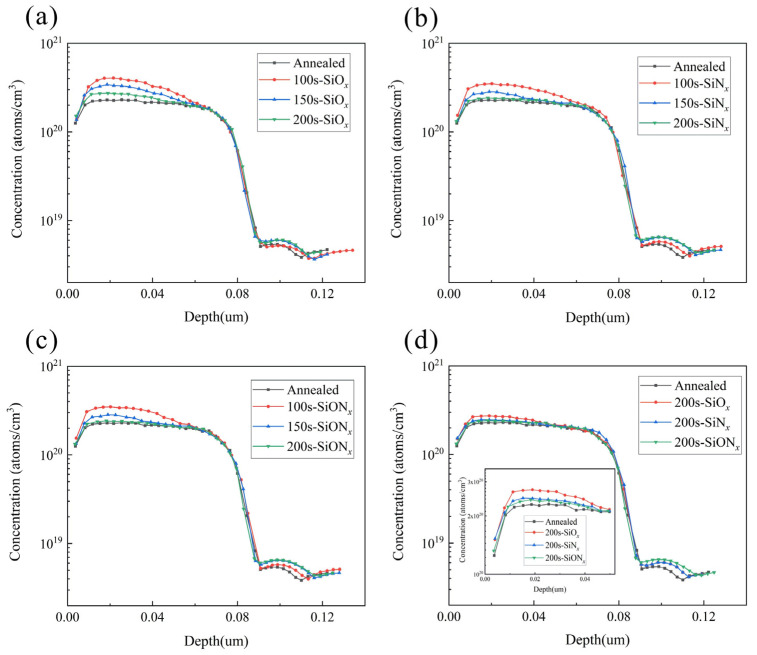
ECV profiles of light diffusion zones with different masks and times: (**a**) SiO*_x_* groups; (**b**) SiN*_x_* groups; (**c**) SiO*_x_*N*_y_* groups; (**d**) 200 s for three mask layers.

The inhibitory effects of various mask structures and mask deposition durations in the three-step laser method on the advancement of P atoms deposition (ECV profiles) are illustrated in Figure 8, while Table 2 presents the peak concentrations of P atoms doping. The measured P atoms concentration after direct annealing in the lightly doped zone is determined to be 2.30 × 10^20^ atoms/cm^3^. It is observed that both SiO*_x_* depositions for 100 s and 150 s exhibit weak blocking effects on P atoms during annealing deposition in the lightly doped zone, failing to effectively maintain the P concentration within this region. However, a good blocking effect against P atoms entering the wafer is achieved with a SiO*_x_* deposition time of 200 s. The peak concentration of P atoms only increases by merely 4.3 × 10^19^ atoms/cm^3^ for this case, whereas for SiN*_x_* at a deposition time of 150 s, it increases by just 5.4 × 10^19^ atoms/cm^3^, and for SiO_x_N*_y_*, it increases by merely 3.6 × 10^19^ atoms/cm^3^. The deposited SiN*_x_* and SiO*_x_*N*_y_* layers at a deposition time of 150 s demonstrate an effective blocking effect on P atoms driving in, while even stronger blocking effects are observed by their respective depositions at 200 s. Considering the imperative for efficient mass production, it is crucial to minimize the process time of the mask layer to ideally within 200 s. To ensure comparability in our study, we standardized the deposition time of the mask layer to 200 s. According to the differential blocking effects of various mask layers on P atoms, SiO*_x_* exhibits the weakest blocking effect, followed by SiN*_x_* and SiO*_x_*N*_y_*. Both SiN*_x_* and SiO*_x_*N*_y_* demonstrate similar blocking effects. Considering the mask layer structure, deposition mask time, and laser power, we designed the experimental groups as presented in Table 2. Additionally, we designed the L1 group with a low doping concentration of non-SE and the H1 group with a high doping concentration of non-SE, respectively. To compare the SE groups using the four-step methods, we also designed the SE groups using the four-step methods with the laser powers for F1–F3 were set at 15 W, 20 W, and 25 W, respectively.

**Figure 9:**
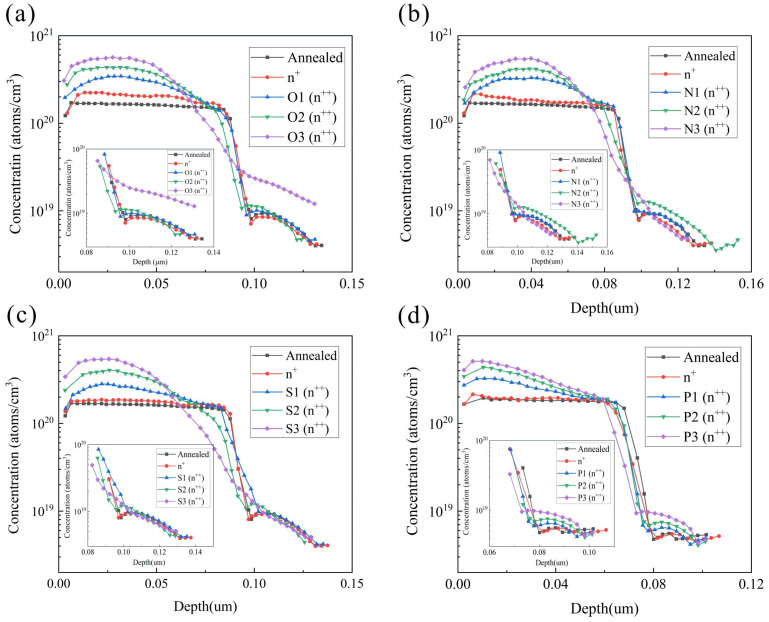
ECV profiles of (**a**) SiO*_x_* groups, (**b**) SiO*_x_*N*_y_* groups, (**c**) SiN*_x_* groups, and (**d**) four-step method groups.

In order to facilitate the investigation of doping concentration changes in the laser region P atoms, we employed a 4 × 4 cm^2^ square laser for experimentation. To avoid discrepancies between wafers, we put the laser square in the position adjacent to the same piece of wafer. The ECV profiles obtained after annealing in different SE regions are depicted in Figure 9. It is observed that varying laser powers significantly influence ECV profiles types, with higher power lasers resulting in steeper emitter profiles and increased doping depths [38]. Elevating the laser power leads to the additional diffusion of phosphorus atoms from the phosphorus emission layer into silicon, thereby forming a highly doped region [38]. The depth of diffusion is typically correlated with the mass of the SiO*_x_* film and the annealing temperature [19]. In the three-step method, the concentration of the shallow surface is driven in by the laser. After opening the mask layer, the wafer is deposited and subsequently driven in. Due to oxidation at 850 °C, the P atoms in the shallow surface precipitate out, resulting in the ECV profiles becoming a parabola. The higher the laser power, the higher the peak concentration.

Laser not only affects peak concentration but also significantly influences poly thickness and ECV inflection point concentration near the wafer. In the three-step method, increasing laser power gradually raises the lower inflection point concentration while weakening the tunneling oxide layer blocking effect. At 25 W laser power, no obvious inflection point is observed in the ECV profiles, and instead it appears smooth. This indicates that the tunneling oxide layer fails to effectively block P atoms from entering into the wafer, and its passivation ability weakens accordingly. Additionally, at 25 W laser power level, the partial ablation of poly occurs making it impossible to determine Poly-Si layer thickness from ECV profiles alone. Figure 9d illustrates the ECV profiles of the four-step method, where peak concentration closely aligns with surface due to mutual diffusion between PSG and Poly-Si [48].

The peak concentrations and inflection point concentrations of various experimental conditions are presented in Table 3. It is observed that the inflection point concentration exhibits an increasing trend with the augmentation of laser power. However, when the laser power reaches 25 W, the inflection point becomes less discernible. The primary cause lies in the excessive infiltration of phosphorus atoms into the wafer through the tunnelling oxide layer, thereby compromising the passivation of SiO*_x_*/Poly-Si(n^+^) and subsequently exerting a direct impact on the solar cells’ electrical performance. In the four-step method, the peak concentration of 15 W is increased from 2.15 × 10^20^ atoms/cm^3^ to 3.25 × 10^20^ atoms/cm^3^, resulting in a net increase of 1.10 × 10^20^ atoms/cm^3^ in concentration. Furthermore, when the laser power is raised to 20 W, an additional increase of 1.13 × 10^20^ atoms/cm^3^ is observed. However, due to limited P atom availability in PSG, increasing the laser power to 25 W only leads to a marginal increment of 7.5 × 10^19^ atoms/cm^3^ compared to that at 20 W.

### 3.3. R_sheet_ of Each Experimental Group

**Figure 10:**
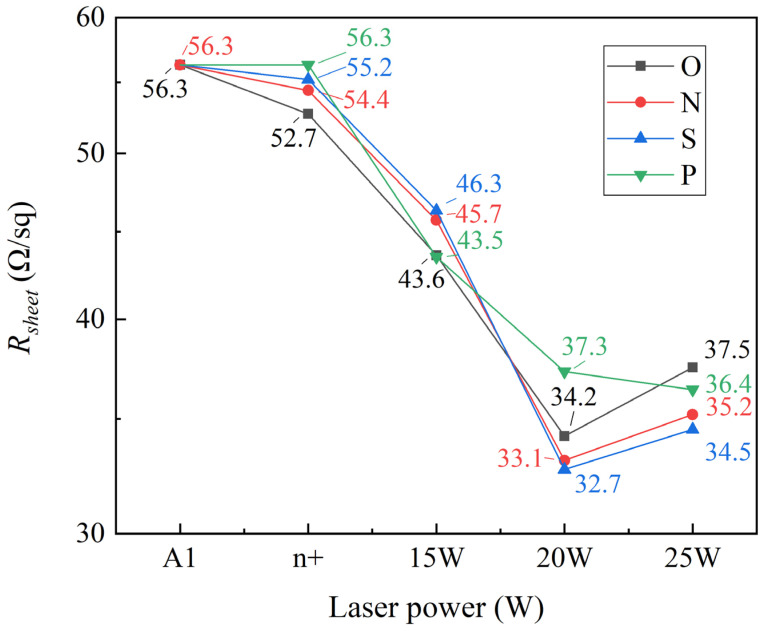
*R_sheet_* of different experimental groups.

Since the formation of a selective emitter enhances the current collection characteristics of the PN junction, the improved *FF* in SE can be attributed to an enhancement in *R_s_* resulting from improved electrode-emitter contact and reduced *R_sheet_* [49]. The *R_sheet_* of each experimental group is illustrated in Figure 10. In the lightly doped region, the *R_sheet_* of the SiO*_x_* mask decreased from 56.3 Ω/sq to 54.4 Ω/sq, followed by the SiN*_x_* group and then the SiO*_x_*N*_y_* group. The blocking ability of the 200 s deposited mask layer against P atoms deposition and diffusion was found to be in the order of SiO*_x_* < SiN*_x_* < SiO*_x_*N*_y_*. These findings are consistent with our ECV testing results in the lightly doped region. However, the *R_sheet_* of the four-step method is considered to be constant due to the simultaneous oxygenation and deposition of P atoms using low temperature precipitation at 750 °C. At this temperature, we believe that there is no driving in effect, and P atoms are solely deposited on the surface. SiO*_x_* can maintain continuity at 750 °C [50]. However, due to the PSG’s blocking action and the phosphorus absorption effect, it becomes challenging for P atoms to drive in or separate out.

When employing 15 W laser power, the three-step laser method only reduces the thickness of the mask layer in the laser region, resulting in a certain level of blocking effect. Conversely, the four-step laser method directly introduces low-temperature deposited P-source into the wafer with a weaker blocking effect. Consequently, at 15 W power, the *R_sheet_* decline is most pronounced for the four-step laser method compared to the three-step laser method. However, as the laser power increases to 20 W, the phenomenon of partial laser film opening to poly is visible in the three-step laser method. As the deposition rate of SiO*_x_* was the slowest, the laser was switched on the most, and partial poly was dissolved. Although the square resistance decreased, the decrease rate was smaller than that of the SiN*_x_* and SiO*_x_*N*_y_* groups. There is no significant difference between the SiN*_x_* and SiO*_x_*N*_y_* groups. From 15 W to 20 W, the wafer *R_sheet_* of the SiO*_x_* group decreased by 9.4 Ω/sq, the *R_sheet_* of the SiN*_x_* group decreased by 10.5 Ω/sq, and the *R_sheet_* of the SiO*_x_*N*_y_* group decreased by 13.6 Ω/sq. When the laser power increases from 15 W to 20 W, the *R_sheet_* continues to decrease with the increase of laser power in the four-step laser method, but the rate of decrease is less than that of the three-step laser method. After the increase of laser power in the three-step laser method, more P atoms are driven into the wafer after the mask is opened, but the amount of P atoms in the PSG was limited. As the laser power increases to 25 W, the *R_sheet_* of the four-step method continues to decrease to 36.4 Ω/sq, slightly surpassing the *R_sheet_* observed with 20 W laser power in the three-step method. Moreover, the 25 W laser power of the three-step method ablates part of the a-Si(n), so the *R_sheet_* of the three-step method shows an increasing trend, and the increase of amplitude is similar. Considering that the SiO*_x_*, SiO*_x_*N*_y_*, and SiN*_x_* mask methods adhere to the same principles under varying power conditions, we exclusively selected the S2 and N2 groups for a comparative analysis.

### 3.4. J_0_, iV_oc_ and τ_eff_

**Figure 11:**
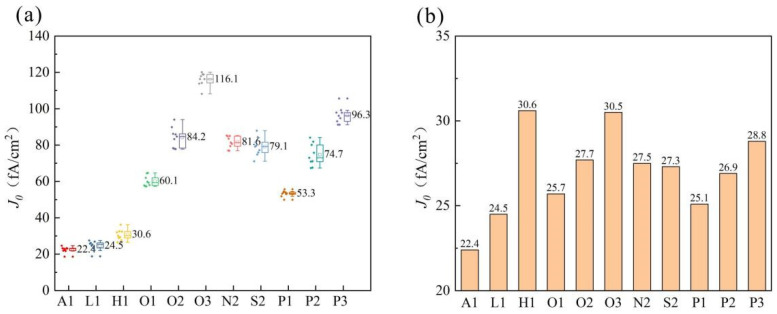
(**a**) *J*_0_ of the full-face laser wafer; (**b**) *J*_0_ calculated from the average value.

In order to compare the conventional process with P-SE, we prepared two experimental groups of concentrations for comparison. The first group is a low concentration group (L1 group) with a peak concentration of approximately 2.0 × 10^20^ atoms/cm^3^. The second group consisted of high concentration samples (H1 group) with a peak concentration of 4.0 × 10^20^ atoms/cm^3^. The rear side of the wafer has 130 grid lines with an actual printed line width of 35 ± 3 μm. Our laser has a spot width of 120 μm, and the grid graphic incorporates a half-piece design to prevent breakage of the grid lines. Based on the laser used for the grid graphic, we calculated that the laser area covers approximately 8.6%. We prepared the non-SE group (SE group light doping zone concentration), L1 group, H1 group, and full-surface laser group following the experimental process in Figure 11b. Before printing and then firing, a *J*_0_ test was carried out. The results are shown in Figure 11.

The *J*_0_ is the current density of recombination, which reflects the strength of the recombination. In order to improve the efficiency of the solar cell, it is necessary to reduce the value of *J*_0_ as much as possible. The *J*_0_ value of the entire wafer after laser treatment is denoted as *J*_0*t,laser*_. The *J*_0_ value of the front and rear sides of the full-face laser on the rear side of the wafer is represented as *J*_0*t,full*_. The sum of the *J*_0_ values for both the front and rear sides of the wafer after direct annealing is expressed as *J*_0*t,annealed*_. The *J*_0_ value of the heavily doped region on the rear side of the wafer is indicated by *J*_0*,finger*_.The lightly doped region on the rear side of the wafer is denoted as *J*_0*,light*_. The front area’s *J*_0_ value is represented by *J*_0*,front*_. *S_laser_* represents the *J*_0_ value in the laser area, while *S_wafer_* denotes its corresponding film area. For calculating different experimental lasers’ respective values of *J*_0*t,laser*_, the following formula is used:

*J*_0*t,laser*_ = *J*_0*,finger*_ + *J*_0*,light*_ + *J*_0*,front*_ = (*J*_0*t,full*_ − *J*_0*t,annealed*_) × (*S_laser_*/*S_wafer_*) + *J*_0*t,annealed*_

After calculation, the *J*_0*t,lase*_*_r_* values are shown in Figure 11b. *J*_0_ increases with the increase of doping concentration, whether it is the three-step method or the four-step method; *J*_0_ increases with the increase of laser power. In laser groups, due to an only 8.6% laser region and a 91.4% light doping region, the comprehensive *J*_0_ is between the L1 and H1 groups. The *J*_0_ of the three masks is very close. Under the same laser power conditions, the *J*_0_ of the four-step method is lower than that of the three-step method.

**Figure 12:**
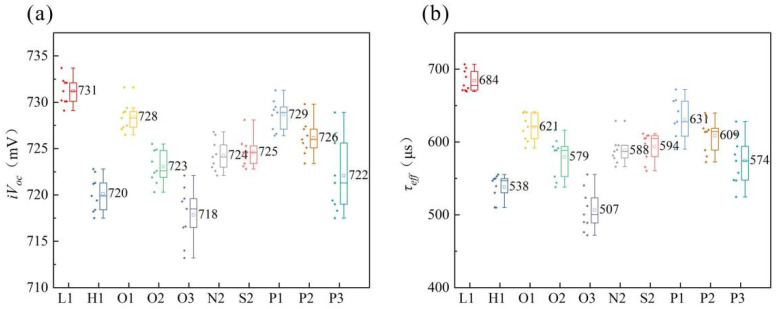
(**a**) *iV_oc_* and (**b**) *τ_eff_* of the precursors.

We conducted direct firing and testing of the wafers prior to printing in order to obtain *iV_oc_* and *τ_eff_* (effective carrier lifetime), as illustrated in Figure 12. Both *iV_oc_* and *τ_eff_* exhibit consistent trends. The L1 group achieved the highest *iV_oc_* at 731 mV, surpassing all the other groups. In contrast, the highly concentrated H1 group exhibited a lower *iV_oc_* of only 720 mV. In the laser groups, the *iV_oc_* of the O3 group with only 25 W is lower than that of the H1 group, while all the other groups exhibit higher *iV_oc_* values compared to the H1 group. Although the peak concentrations in the doped region of the O2, S2, and N2 groups is higher than that in the H1 group, due to its limited coverage (8.6% of the rear-side surface), the lightly doped region exhibits a lower concentration (91.4% of the rear-side surface) compared to that in the H1 group. Additionally, the mask layer in the lightly doped region fails to completely obstruct the P atoms, resulting in a similar concentration of the lightly doped region in the SE group compared to the L1 groups. Consequently, laser damage occurs within this region, serving as a recombination center for carriers. As a result, both *iV_oc_* and *τ_eff_* are lower in the SE group than those observed in the L1 group with a larger reduction range.

In order to enhance the concentration of the contact region, the F1–F3 groups employed a four-step laser method to deposit P atoms ahead of the laser, while utilizing a four-step laser method resulted in a thicker PSG mask layer, thereby minimizing damage compared to the three-step laser method. Under identical laser power conditions, *iV_oc_* and *τ_eff_* exhibit higher values than those achieved with the three-step laser method. Increasing the laser power led to elevated recombination rates, indicating that high-power lasers can induce defect formation (such as cracks and displacements). However, when operating at powers below 20 W, even graphics with less than 9% coverage display favorable *iV_oc_* and *τ_eff_*.

### 3.5. Specific Contact Resistivity

**Figure 13:**
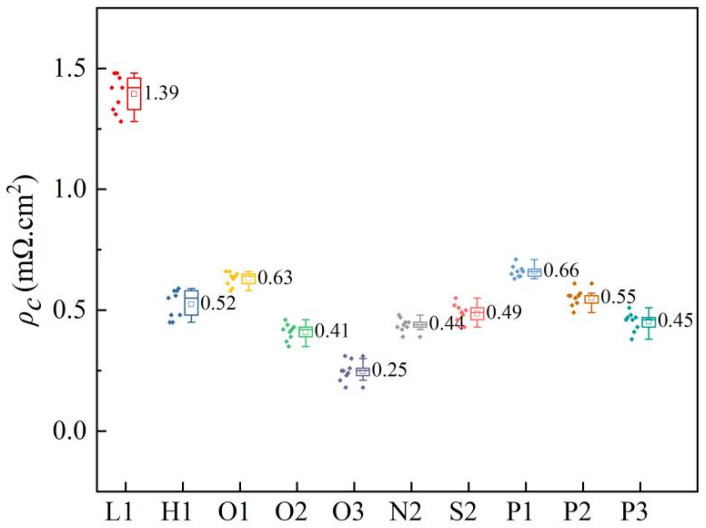
Specific contact resistivity of the rear side for each experimental group.

The significant increase in *FF* can be attributed to the presence of a heavily doped surface beneath the silver-metal contacts, which effectively drives a limited number of carriers by reducing surface recombination through field-effect passivation [49]. The specific contact resistivity of the rear side for each experimental group is illustrated in Figure 13. As the concentration of the contact area increases, there is a gradual decrease in the specific contact resistivity of the rear side. Among all groups, the O3 group exhibits superior performance with a remarkable specific contact resistivity value of only 0.25 mΩ/cm^2^. Notably, the concentration of P atoms within the contact area emerges as a critical factor influencing the observed specific contact resistivity. The specific contact resistivity decreases as the P atoms doping concentration in the contact region increases. Under the 20 W laser condition, both the three-step and four-step laser methods outperform the H1 high doping contacts. By combining *iV_oc_* and *τ_eff_* in Figure 12, it is observed that at 20 W, the *iV_oc_* and three-step laser method exhibit superior performance compared to the high-doping concentration group, thus achieving a selective emitter effect in this study. The low doping concentration group achieved a higher *iV_oc_* but compromised on contact quality.

### 3.6. Electrical Performance

The electrical performance data of each experimental group are presented in Table 4. The low doping concentration group (L1) exhibits the highest *V_oc_*, albeit at the expense of a significant reduction in *FF*, resulting in a solar cell efficiency of 24.68%. On the other hand, the high doping concentration group (H1) achieved higher *FF* by sacrificing *V_oc_*, leading to an efficiency of 24.65%, which is marginally lower than that of the L1 group by 0.03%. Among the different laser power groups of SiO*_x_*, the O1 group exhibits a higher *V_oc_,* which is 5.2 mV greater than that of the H1 group. Additionally, it demonstrates significantly improved *FF* compared to the L1 group and achieves a solar cell efficiency that is 0.04% higher than that of the L1 group. Despite Group O2 having a slightly compromised *V_oc_* compared to Group O1, it showcases further enhancement in *FF* and attains the highest solar cell efficiency of 24.85%, surpassing Group L1 by 0.17%. The implementation of a selective emitter enhances the current collection characteristics of the PN junction, while the enhancement in *FF* can be attributed to an improvement in series resistance (*R_s_*) [49]. However, when the laser power is increased to 25 W, Group O3 exhibits a significant decrease in *FF* and *J_sc_*, resulting in a solar cell efficiency of only 23.72%, which is 0.96% lower than that of Group L1. This decline can primarily be attributed to severe damage inflicted on Poly-Si(n^+^) due to excessive laser power, leading to substantial surface damage and consequently causing notable reductions in *V_oc_* and *J_sc_*. It can be seen from Figure 9a that the laser power of 25 W reduced the thickness of Poly-Si, which resulted in the decrease of *FF*. At the same time, too many P atoms entered the Si bulk through the tunnel oxide layer, the tunneling effect of the solar cell weakened, and a large recombination was brought about, all of which resulted in the decrease of *V_oc_* and *J_sc_*. As shown in Figure 12a, *iV_oc_* decreased significantly, and there was a corresponding relationship between *iV_oc_* and *V_oc_*. From the efficiency formula of the solar cell, *E_ff_* = *V_oc_* × *J_sc_* × *FF/*1000 × 100%, it can be seen that the *E_ff_* of the solar cell is proportional to *V_oc_*, *J_sc_*, and *FF*, and the *V_oc_*, *J_sc_*, and *FF* of the O3 group are significantly reduced than those of the O2 group, bringing about a significant decline in *E_ff_*. The laser power of the S2 and N2 groups are equivalent; however, there exists a slight disparity in the ECV profiles. The discrepancies in *V_oc_*, *J_sc_*, and *FF* are minimal, with only a 0.02% variance observed in the efficiency of solar cells. Furthermore, the difference in efficiency of solar cells between the N2 group and O2 group is merely 0.1%. Notably, the *V_oc_* of the O2, S2, and N2 groups gradually diminishes primarily due to light doping within this region. Additionally, distinct mask layers exhibit varying abilities to impede P diffusion into Poly-Si. The four-step method adheres to a consistent rule, with the P2 group exhibiting superior efficiency. As a result of direct laser doping, the concentration of the non-contact region remains unchanged and is lower compared to that achieved by the three-step method. Consequently, higher *V_oc_* and *J_sc_* values can be attained in comparison to those obtained using the three-step method. We found that the *FF* improvement in the O2 group is larger than that in the P2 group. This could potentially be attributed to a higher P atoms doping concentration in the O2 group as opposed to the P2 group, primarily driven by externally deposited P atoms and further facilitated by elevated temperatures, thereby enabling the attainment of a higher *FF* in Group O2. The surface damage in Group P3 occurred due to the high laser power, resulting in a slight decrease in *V_oc_* and *J_sc_*. However, the improvement of *FF* is insufficient to compensate for this decline, leading to an overall reduction in the efficiency of solar cells. Finally, the efficiency of the P3 group is close to that of the P1 group.

## 4. Discussion

The rear-side laser has a significant impact on the *V_oc_*, *J_sc_*, and *FF*, which has an impact on the efficiency of solar cells. The main factors affecting the *V_oc_* are as follows:

$$Voc=\frac{kT}{q}\ln\frac{Jsc+J01}{J01}\approx \frac{kT}{q}\ln\frac{Jsc}{J01}$$

**Figure 14:**
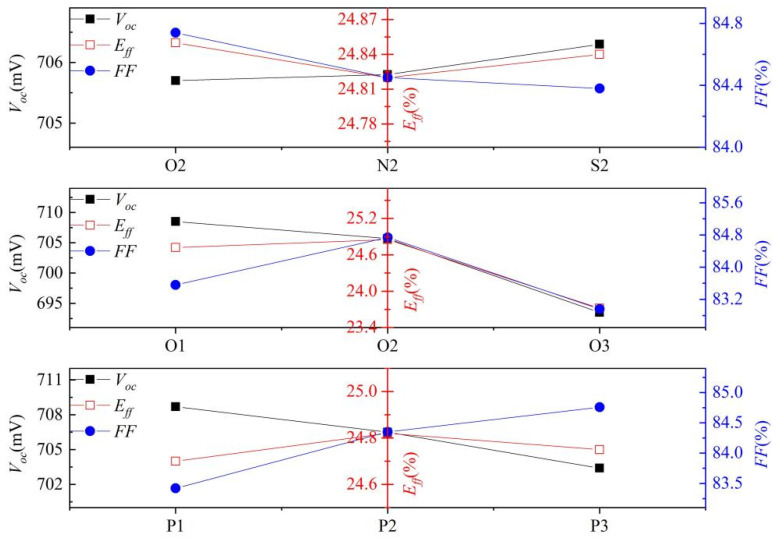
Comparison of *V_oc_*, *FF* and *E_ff_* of three masks groups, three-step groups, and four-step groups.

In this equation, *J_sc_* ≫ *J*_01_, a strong correlation between *V_oc_* and the dark current is observed, encompassing reverse saturation current, thin layer leakage current, and bulk leakage current. During the process of achieving a selective emitter on the rear side, certain damages are formed that act as recombination centers by trapping electrons and holes for subsequent recombination. The *V_oc_* of the SiO*_x_*, SiN*_x_*, and SiO*_x_*N*_y_* groups exhibit minimal variation. As shown in Figure 14, the *V_oc_* gradually decreases with increasing laser power for the three-step and four-step SE groups. In the 15 W laser group (O1), only a partial vaporization of the mask layer occurred without any damage to the poly layer. The main gasification of the mask layer still occurred in the 20 W laser group (O1), while causing some destruction to the Poly-Si layer. However, when the laser power was increased to 25 W (O3 group), a further decrease in *V_oc_* was observed, primarily due to more significant damage inflicted by the laser on the poly layer. Consequently, compared to the O1 group, the O2 group exhibited a smaller reduction in *V_oc_*, whereas the O3 group demonstrated a greater decrease than that of the O2 group. In the four-step method, despite an increase in laser power, the declining trend of *V_oc_* remained consistent. This can be attributed to the relatively thicker PSG layer compared to the SiO*_x_* mask layer and the minimal damage caused by a 25 W laser on Poly-Si.

The *FF* has a direct relationship with contact resistance, and the contact resistance is directly affected by the concentration of P atoms in the contact region [49]. Selective emitter formation enhances the current collection characteristics of PN junctions. The P atoms doping concentrations of the SiO*_x_*, SiN*_x_*, and SiO*_x_*N*_y_* mask layers are comparable. Hence, there is no significant difference in the *FF* values. Moreover, due to similar *V_oc_* values among these three groups, their efficiencies also exhibit close proximity. 

In the three-step selective emitter groups, the *V_oc_* exhibits a gradual decline with increasing laser power, while the *FF* and *E_ff_* display an initial increase followed by a subsequent decrease. Amongst these groups, the 20 W O2 group demonstrates the highest efficiency, whereas the 25 W O3 group exhibits significantly lower efficiency compared to both the O1 and O2 groups. 

In the four-step selective emitter groups, the *FF* shows a progressive increase with increasing power; however, the overall *E_ff_* also showcases an initial rise followed by a subsequent decline due to the combined influence of *V_oc_*, *J_sc_*, and *FF*. The P2 group achieves the highest efficiency, while the P1 group has the lowest efficiency. Notably, there is minimal disparity in the efficiency of solar cells among the three laser groups indicating their higher tolerance towards laser irradiation as compared to that observed in the four-step method.

The increase in annealing temperatures leads to a higher kinetic energy in dopant atoms, while SiO*_x_* is degraded at elevated temperatures, resulting in an augmentation of pinhole formation and increased bulk recombination [51] and thereby reducing passivation efficiency. The annealing temperature not only influences the density and integrity of SiO*_x_* but also significantly impacts the crystallization rate of Poly-Si (χc). The χc value of silicon can be increased by raising the annealing temperature [52]. However, it should be noted that χc does not exhibit a linear relationship with temperature due to its susceptibility to various factors such as film composition and stress release. Moreover, achieving optimal passivation results requires a delicate balance between multiple mechanisms. Therefore, a higher Poly-Si value of χc does not necessarily guarantee superior passivation performance.

**Figure 15:**
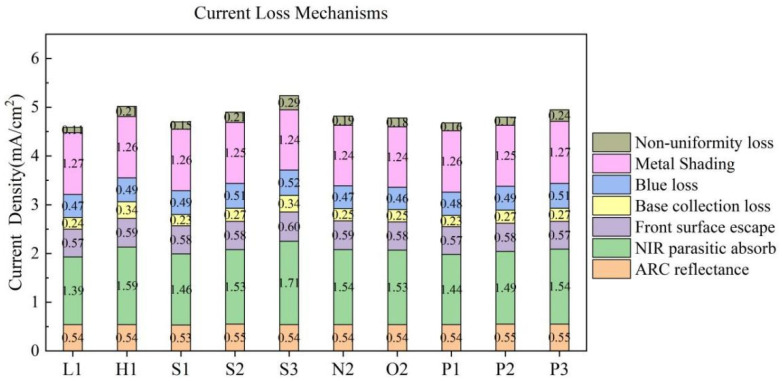
Current loss analysis of each experimental group.

The current loss analysis of each experimental groups is presented in Figure 15. The influence mechanism of a short-circuit current is relatively complex, which is comprehensively affected by surface area recombination, metal recombination, contact properties, etc. The n-TOPCon solar cells mitigate compound loss in the base layer by tunneling through the oxide layer and implementing Poly-Si bulk passivation.

The primary differences among the groups are attributed to NIR parasitic absorption, base collection loss, and non-uniformity loss. Near-infrared light is partially reflected by the rear, and some of it is absorbed and lost, resulting in rear parasitic absorption loss. Different laser conditions lead to varying degrees of passivation in the laser region, which affect near infrared parasitic absorption losses. Higher laser power results in greater NIR parasitic absorption loss. The parasitic absorption loss of near-infrared is greater at high doping concentrations compared to low doping concentrations. The near-infrared parasitic absorption loss follows the same trend as the base collection loss and non-uniform loss, which are also influenced by the quality of rear-side passivation. Despite an increase in laser power leading to a decrease in specific contact resistance and an improvement in carrier collection ability, there is a simultaneous increase in base collection loss. Amongst all groups, the H1 group exhibits the highest base collection loss primarily due to its high doping concentration on the rear side. On the other hand, the O3 group experiences decreased bulk passivation mass resulting from the high laser energy causing some P atoms to penetrate through the tunnel oxidation layer into the wafer.

Both the three-step and four-step laser methods can enhance the efficiency of n-TOPCon solar cells, with the former reducing one step in industrial production. The various mask structures of the three-step method effectively block the light-doped region P atoms to achieve a selective emitter. The three-step method is simpler and more effective but not more stable. Because the P atoms of the three-step method are deposited at 920 °C for 7 min, the high temperature increases the diffusion rate of P atoms, which forms a severe wrap-round of P atoms. The severe wrap-round of P atoms forms BPSG with BSG at the front, which is difficult to clean. The four-step method only needs to deposit the P atoms at a low temperature because there is also a step of oxidation annealing for 45 min, which is enough to form good PSG that is protective of the wafer. The corrosion in the cleaning process needs to be strictly controlled, and the four-step method provides better control. The large-scale production additives and cleaning processes currently focus on SiO*_x_* and PSG, with better coordination of the SiO*_x_* mask layer. However, the mask layer containing N also presents an environmental issue of N emission, so, we recommend the SiO*_x_* mask. The efficiency of SiO*_x_* is the highest, which is mainly due to the best matching of 20 W laser power, 200 s SiO*_x_*, the amount of deposited P atom, etc. Due to the better blocking effect of SiN*_x_*, the optimal power matching condition may be above 20W but below 25 W. The same effect can be achieved in the four-step method. 

The precise control of laser power during mask opening yields excellent comprehensive electrical properties without damaging Poly-Si. Although laser technology is a very attractive approach for a large number of applications in silicon photovoltaic manufacturing, its application requires a significant amount of fine-tuning of laser parameters to reduce the harmful effects that accompany laser processing. In particular, laser processing can adversely affect device performance by reducing minority carrier lifetime [41,53,54,55], increasing leakage current [41,42,56], and reducing carrier transport [57]. All of these depend on the surface preparation, the pre-existing dielectric pile, and the laser conditions (pulse duration and energy density) of the application. The optimization direction of the rear side of n-TOPCon solar cells can be achieved through the experimental method employed in this study, enabling the preparation of a-Si without doping or with a lower concentration of doping to achieve a poly-finger structure on the rear side. Currently, mass-produced LECO technology [58] utilizes full-face laser treatment, which not only enhances contact on the front side but also improves contact performance on the rear side. This approach enables excellent contact performance at low doping concentrations and allows for lower doping concentrations in non-contact areas on the rear side, resulting in higher overall electrical properties. The future optimization direction of n-TOPCon solar cells also encompasses the reduction in poly layer thickness to mitigate near parasitic absorption losses [59,60]. Furthermore, the prospective technological advancements include double-sided TOPCon technology, poly-finger technology, and ultimately integrating BC with TBC technology.

## 5. Conclusions

The rear-side selective emitter technique significantly impacts the passivation and contact properties of n-TOPCon solar cells. Both the three-step and the four-step laser methods can effectively reduce the specific contact resistivity at the rear-side surface. In the three-step method, among the three mask layers deposited for 200 s, the blocking effect on phosphorus in the non-contact region followed the order: SiO*_x_* < SiN*_x_* < SiO*_x_*N*_y_*. Considering current mass production cleaning conditions, SiO*_x_* is recommended as the optimal choice for mass production without introducing nitrogen elements. In terms of stability, the four-step method exhibits higher performance compared to the three-step method. However, in terms of comprehensive electrical properties, the three-step method can achieve comparable results to the four-step method. The O2 group demonstrated the highest efficiency (24.85%), with a comprehensive *V_oc_* of 2.4 mV higher than that of the high doping concentration group (H1 group). Additionally, it exhibits an increase in *FF* by 1.88% compared to the low doping concentration group (L1 group), resulting in a final efficiency that is 0.17% higher than the L1 group and 0.20% higher than the H1 group. Compared with Group O2 (20 W), its *V_oc_* decreases by 12.2 mV, leading to a substantial decline in the solar cell’s electrical performance with an efficiency of only 23.72%. In the four-step method, the group with a laser power of 20 W (P2 group) achieved the highest efficiency (24.82%). The *V_oc_* is 3.2 mV higher than that of the H1 group, while the *FF* is 1.49% higher than that of the L1 group. Moreover, the overall efficiency is found to be 0.14% higher than that of the L2 group and 0.17% higher than that of the H1 group. Notably, in comparison to both the L1 and H1 groups, each laser condition group demonstrates significant improvements within this four-step method study, thereby providing valuable guidance for enhancing n-TOPCon solar cells’ back technology during mass production.

## Figures and Tables

**Table 1:** Different experimental groups of P-SE process.

Groups	L1	H1	O1	O2	O3	N1	N2	N3	S1	S2	S3	P1	P2	P3
Laser/w	—	—	15	20	25	15	20	25	15	20	25	15	20	25
mask	—	—	SiO*_x_*			SiN*_x_*			SiO*_x_*N*_y_*			PSG	PSG	PSG
Steps	—	—	Three									Four		

**Table 2:** Peak concentrations of light doping regions in different mask layer groups.

Mask	100 s (Atoms/cm^3^)	150 s (Atoms/cm^3^)	200 s (Atoms/cm^3^)
SiO*_x_*	4.05 × 10^20^	3.42 × 10^20^	2.73 × 10^20^
SiN*_x_*	3.46 × 10^20^	2.69 × 10^20^	2.47 × 10^20^
SiO*_x_*N*_y_*	3.49 × 10^20^	2.84 × 10^20^	2.42 × 10^20^

**Table 3:** Peak concentrations of ECV profiles at different conditions.

Mask	n^+^ (Atoms/cm^3^)	15 W (Atoms/cm^3^)	20 W (Atoms/cm^3^)	25 W (Atoms/cm^3^)
SiO*_x_*	2.24 × 10^20^	3.44 × 10^20^	4.35 × 10^20^	5.66 × 10^20^
SiN*_x_*	2.18 × 10^20^	3.28 × 10^20^	4.19 × 10^20^	5.50 × 10^20^
SiO*_x_*N*_y_*	1.87 × 10^20^	2.82 × 10^20^	4.06 × 10^20^	5.43 × 10^20^
PSG	2.15 × 10^20^	3.25 × 10^20^	4.38 × 10^20^	5.13 × 10^20^

**Table 4:** Electrical performance parameters of each experimental group.

Groups	*V_oc_* (mV)	*J_sc_* (mA/cm^2^)	*R_s_* (mΩ)	*FF* (%)	*E_ff_* (%)
L1	711.4	41.87	5.37	82.86	24.68
H1	703.3	41.34	3.48	84.77	24.65
O1	708.5	41.76	4.63	83.56	24.72
O2	705.7	41.56	3.93	84.74	24.85
O3	693.5	41.22	3.65	82.96	23.72
N2	705.8	41.64	4.01	84.45	24.82
S2	706.3	41.68	4.26	84.38	24.84
P1	708.7	41.78	4.87	83.42	24.70
P2	706.5	41.65	4.12	84.35	24.82
P3	703.4	41.41	3.76	84.76	24.69

## Data Availability

Data are contained within the article.
